# The proteomic response in glioblastoma in young patients

**DOI:** 10.1007/s11060-014-1474-6

**Published:** 2014-05-18

**Authors:** Ruth F. Deighton, Thierry Le Bihan, Sarah F. Martin, Martin E. Barrios-Llerena, Alice M. J. Gerth, Lorraine E. Kerr, James McCulloch, Ian R. Whittle

**Affiliations:** 1 Department of Clinical Neurosciences, Western General Hospital, Edinburgh, EH4 2XU UK; 2 Centre for Integrative Systems Biology, University of Edinburgh, Edinburgh, EH9 3JD UK; 3 Centre for Cognitive and Neural Systems, University of Edinburgh, Edinburgh, EH8 9JZ UK; 4 School of Biomedical Sciences, University of Edinburgh, Hugh Robson Building, Edinburgh, EH8 9XD UK

**Keywords:** Clinical proteomics, Glioblastoma, Patient age

## Abstract

Increasing age is an important prognostic variable in glioblastoma (GBM). We have defined the proteomic response in GBM samples from 7 young patients (mean age 36 years) compared to peritumoural-control samples from 10 young patients (mean age 32 years). 2-Dimensional-gel-electrophoresis, image analysis, and protein identification (LC/MS) were performed. 68 proteins were significantly altered in young GBM samples with 29 proteins upregulated and 39 proteins downregulated. Over 50 proteins are described as altered in GBM for the first time. In a parallel analysis in old GBM (mean age 67 years), an excellent correlation could be demonstrated between the proteomic profile in young GBM and that in old GBM patients (r^2^ = 0.95) with only 5 proteins altered significantly (*p* < 0.01). The proteomic response in young GBM patients highlighted alterations in protein–protein interactions in the immunoproteosome, NFkB signalling, and mitochondrial function and the same systems participated in the responses in old GBM patients.

**Electronic supplementary material:**

The online version of this article (doi:10.1007/s11060-014-1474-6) contains supplementary material, which is available to authorized users.

## Introduction

Patients diagnosed with Glioblastoma (GBM, WHO-IV) have extremely poor median survival times, despite modern microsurgery, chemoradiotherapy, reoperation and experimental therapies [1–4]. To improve GBM treatment and patient median survival times, fresh insight into the molecular pathogenesis of GBM is essential. Proteomics can define molecular pathways and cellular functions altered in GBM [5]. Genomic studies, although important, are limited by the fact that normal, upregulated or mutated genes may not be transcribed for a number of epigenetic reasons [6]. Multiple discrepancies between mRNA and proteomic expression profiles in differential analyses of gliomas highlight the importance of studying protein expression [7].

Age is a powerful individual prognostic indicator [8–10]. Long term survivors of GBM are invariably younger patients [8–10], and in one randomised clinical trial, median survival for GBM cohorts aged <45 years was 48 weeks compared to 19 weeks for those >65 years; and at 18 months 23 % of the younger cohort was alive compared to 3 % of the older cohort [8]. Numerous randomised controlled trials and hospital series have excluded differences in access to health care as the cause for this differential outcome [1, 9–12].

The biological basis of this powerful age-related effect is not understood. The histological features of GBM, cellular proliferative indices, epidermal growth factor receptor amplification and p53 expression are very similar irrespective of age [9]. Although younger patients are more likely to have secondary GBM than the elderly [3, 9, 13], there is no difference in outcomes between primary and secondary GBM once the diagnosis is made and patients have been aged matched with controls [13]. Although genetic differences associated with short survival (6q loss, 10q loss, 19q gain, sodium ion channel mutations) and longer survival (TP53 mutations and the combination of LOH1p and LOH19q, MGMT status, mutation of IDH1) have been identified, these differences have not, with the exception of IDH1 mutations where the mutation occurred in younger patients, been analysed with respect to patient age [14–20].

Analyses of GBM samples from older patients has begun to provide a coherent view of the proteomic response in GBM but interpretation is complicated by differences in experimental design and proteomic technology [5]. In this study we provide the first systematic proteomic analysis in young GBM (versus age-matched peritumoural-control brain) to gain insight into the basis of the importance of age on prognosis. For the purpose of comparison, we performed a parallel, contemporaneous study (using the same experimental design and technology) in old GBM.

## Materials and methods

### Clinical material

Glioblastoma and peritumoural-control brain samples were obtained from young (<45 years) and old patients (>60 years) undergoing resective brain tumour surgery (Ethical approval: LREC/2004/4/16). The sampling procedure and clinical details of experimental samples are described in Supplementary Tables 1 and 2 and Supplementary methods.

The experimental group sizes used for the primary proteomic analysis were young GBM (n = 7) and young peritumoural-control (n = 12) (based on a priori power calculations to detect significant changes of ≥35 % with power ≥0.8). Tissue was collected for two comparison groups: old GBM (n = 13) and old peritumoural-control (n = 10). The median co-efficients of variation were similar in each experimental group: young GBM 33.55 %, young peritumoural-control 27.38 %, old GBM 33.99 % and old peritumoural-control 26.39 % (Supplementary Fig. 1).

Total protein extracts were separated by isoelectric point and molecular mass using 2DGE (see Supplementary-methods). 2D-gel images were captured using a FluorChem Image Analyser and aligned in a single study using at least four manual alignment vectors followed by automatic placement of further alignment vectors by the software (~200–400 vectors per gel). The mean protein levels of each protein were analysed using Student’s *t* test (*p* ≤ 0.003, equivalent to *p* ≤ 0.01 with Bonferroni correction factor 3 for each comparison). Significant data are presented in Table 1 (uncorrected for multiple comparisons) and all data are presented in Supplementary Table 3. Protein spots differentially expressed in young GBM versus young peritumoural-control were manually excised and proteins identified using LC–MS [21]. LC–MS runs of each sample were combined using Maxquant, assuming a false positive rate of 0.01 [22]. An identical approach was applied in parallel comparing old GBM versus old peritumoural-control, and young GBM versus old GBM.

**Table 1:** Proteins altered in young GBM

Spot ID	Protein ID	Protein accession number	Young GBM		Old GBM		Main protein function
			Fold change	*p* value	Fold change	*p* value	
746	CKMT1A	P12532	0.32	1.76E−08	0.53	3.95E−05	ATP homeostasis
749	GNB1*	P62873	0.59	2.05E−07	0.63	2.29E−05	GPCR beta subunit
757	DPYSL2	Q16555	0.4	3.94E−07	–	–	Cytoskeletal
798	INA	Q16352	0.36	4.21E−07	0.59	0.00240	Cytoskeletal
271	ALDOA	P04075	0.71	5.48E−07	1.3	0.00034	Glycolysis
310	CRYM	Q14894	0.37	7.79E−07	0.55	9.96E−05	–
768	STMN1	P16949	0.38	8.37E−07	0.59	0.00015	Cytoskeletal
161	GDI2	P50395	0.34	9.28E−07	–	–	–
119	OXCT1	P55809	0.37	9.71E−07	–	–	Lipid metabolism
67	DPYSL2	Q16555	0.45	1.11E−06	0.63	0.00246	Cytoskeletal
760	VDAC2	P45880	0.53	1.28E−06	0.53	1.40E−06	Ion transport
763	GOT1	P17174	0.53	2.09E−06	0.53	1.86E−05	Amino acid metabolism
736	GNB1*	P62873	0.43	2.32E−06	0.47	1.05E−06	GPCR beta subunit
343	NAPB	Q9H115	0.5	2.34E−06	0.55	7.05E−06	Ca^2+^ mediated exocytosis
469	NDUFS3	O75489	2.9	2.83E−06	2.5	3.00E−06	Electron transport
492	C1orf128	Q9GZP4	0.36	3.55E−06	0.5	2.36E−05	unknown
120	OXCT1	P55809	0.48	3.70E−06	0.63	0.00026	Lipid metabolism
451	PNPO*	B4E152	2.1	6.70E−06	–		Pyridoxine biosynthesis
249	TUBB2A	Q13885	0.5	7.44E−06	–		Cytoskeletal
243	–	–	0.59	1.25E−05	0.59	1.29E−05	–
938	MBP	P02686	0.53	1.36E−05	–		Myelin
809	PSAT1	Q9Y617	0.53	1.45E−05	–		Amino acid biosynthesis
84	INA	Q16352	0.33	1.51E−05	0.45	3.21E−05	Cytoskeletal
613	PGAM1	P18669	0.33	1.68E−05	0.55	0.00044	Glycolysis
428	PSME1	Q06323	2.3	1.76E−05	2.0	3.40E−05	Immunoproteosome
734	TUBB2A	Q13885	0.42	1.79E−05	0.48	1.46E−06	Cytoskeletal
785	UCHL1	P09936	0.59	1.89E−05	0.55	9.13E−06	Stabilises free ubiquitin
544	TAGLN3	Q9U115	0.5	2.55E−05	0.5	4.52E−07	Neuronal growth
1046	TUBB2C	P68371	0.45	2.59E−05	0.59	0.00113	Cytoskeletal
774	PDXP	Q96GD0	0.33	2.62E−05	0.5	4.72E−05	Phosphatase activity
794	PRDX3*	P30048	1.9	2.72E−05	–	–	Antioxidant
823	HPRT1	P00492	1.6	3.32E−05	–	–	Purine synthesis
379	VDAC2	P45880	0.71	3.67E−05	–	–	Ion transport
748	NAPG	Q99747	0.45	4.70E−05	0.59	9.04E−06	Vesicle transport
459	UCHL1	P09936	0.77	4.77E−05	–	–	Stabilises free ubiquitin
657	UBE2 N	P61088	2.2	5.20E−05	–	–	Ubiquitination
786	SEPT11	Q92599	0.59	6.54E−05	0.63	0.00129	Vesicle transport
828	PRDX3	P30048	0.66	7.37E−05	0.77	0.00058	Antioxidant
812	PSME2	Q9UL46	2.0	9.5E−05	2.0	0.00169	Immunoproteosome
822	HSPD1	P10809	0.63	9.5E−05	0.63	0.00173	Chaperone
1062	HSPB1	P04792	0.5	0.000106	0.71	0.00219	Chaperone
69	DPYSL2	Q16555	0.66	0.000118	–	–	Cytoskeletal
285	ACOT7	O00154	0.5	0.000124	0.53	2.84E−05	Acetyl-CoA binding
605	MBP*	P02686	0.53	0.000131	–	–	Myelin
116	PHGDH	O43175	0.55	0.000132	0.53	0.00187	Serine biosynthesis
467	GFAP	P14136	2.4	0.000148	–	–	Cytoskeletal
483	DCXR*	Q7Z4W1	2.7	0.000186	–	–	Glucose metabolism
868	UCHL1	P09936	0.66	0.000229	–	–	Stabilises free ubiquitin
276	IDH3A	P50213	0.48	0.000239	0.59	3.65E−06	TCA cycle
916	CKB	P12277	0.66	0.000245	0.66	1.49E−05	ATP homeostasis
487	TPI1	D3DUS9	2.1	0.000251	–	–	Glycolysis
556	PEBP1	P30086	2.7	0.000255	–	–	Intracellular signaling
718	DCD	A5JHP3	0.37	0.000259	0.43	3.06E−06	Phosphatase activity
66	DPYSL2	Q16555	0.63	0.000316	–	–	Cytoskeletal
92	CCT6A	P40227	2.2	0.000391	2.0	0.000454	Protein folding
91	HIST1H4A*	P62805	1.6	0.000479	–	–	Chromatin binding
498	GRB2	P62993	0.63	0.000499	–	–	Signal transduction
273	hCG_2002*	Q59GE1	0.71	0.000548	0.66	6.60E−05	Neuron growth
579	DCD	A5JHP3	2.4	0.000596	–	–	Phosphatase activity
62	DPYSL2	Q16555	1.7	0.000689	–	–	Cytoskeletal
756	ATP6V1E1	P36543	0.71	0.000709	0.63	2.82E−05	Energy metabolism
437	CLIC*	Q9Y696	2.4	0.000737	2.5	0.000155	Ion transport
288	TUBB2A	Q13885	0.66	0.000745	–	–	Cytoskeletal
731	TF*	P02787	1.5	0.000749	–	–	Iron transfer
843	PDIA3	P30101	1.4	0.000751	1.3	0.00263	Protein folding
409	HSPA5	P11012	2	0.000810	1.8	0.00035	Chaperone
488	APOA1*	P02647	0.55	0.000811	0.53	0.00026	Lipid metabolism
270	ALDOA	P04075	0.71	0.000941	0.66	0.00124	Glycolysis
771	GFAP*	P14136	1.8	0.000971	1.7	0.000953	Cytoskeletal
740	HSPB1	P04792	0.66	0.00101	0.71	0.00193	Chaperone
898	GLUD1*	P00367	1.4	0.00107	–	–	Glutamate turnover
966	ATP6V1B2	P21281	0.59	0.00112	–	–	Energy metabolism
263	OvBr SEPT	Q9UHD8	0.66	0.00114	–	–	Cytoskeletal
789	hCG_2002	Q59GE1	0.59	0.00115	–	–	Neuronal growth
801	SEPT11	Q9NVA2	0.63	0.00124	0.66	0.00104	Vesicle transport
154	SEPT11	Q9NVA2	0.71	0.00133	0.63	0.00161	Vesicle transport
28	GPD2	P43304	1.9	0.00149	–	–	Lipid metabolism
207	ACTR1B	P42025	0.63	0.00169	–	–	Cytoskeletal
25	HSPA8	P11142	0.63	0.00187	–	–	Chaperone
1012	SRI	P30626	1.8	0.00191	–	–	Calcium homeostasis
516	GSTP1	P09211	1.3	0.00192	1.2	0.000867	Free radical clearance
342	DKFZp686	P07355	1.4	0.00193	1.9	0.00220	unknown
572	TAGLN3	Q9U115	2.8	0.00200	–	–	Neuronal growth
81	CAT	P04040	1.4	0.00218	–	–	Nucleotide binding
434	GSTO1	P78417	1.5	0.00234	–	–	Glutathione metabolism
444	ACOT7	O00154	1.6	0.00239	–	–	Acetyl-CoA binding
838	PGAM1	P18669	1.6	0.00249	–	–	Glycolysis
876	ALAD*	P13716	1.6	0.00251	–	–	Haeme production
324	TUBB2B	Q9BVA1	0.77	0.00263	–	–	Cytoskeletal
403	GNB1	P62873	0.63	0.00269	–	–	GPCR subunit
829	SNCG	A9XXE1	–	–	0.38	7.58E−09	unknown
945	HIST1H4A	P62805	–	–	1.6	1.79E−05	Chromatin binding
277	ALDOA	P04075	–	–	0.71	3.98E−05	Glycolysis
401	CLIC1	O00299	–	–	0.55	0.000135	Ion transport
1073	UQCRFSL	P0C7P4	–	–	0.66	0.000261	unknown
772	PSMB7	Q99436	–	–	1.5	0.000261	20 s proteosome
564	PEBP1	P30086	–	–	0.71	0.000298	Intracellular signaling
840	MAP2K1	Q02750	–	–	0.71	0.000313	Intracellular signaling
317	LASP1	Q14847	–	–	1.8	0.000322	Cytoskeletal
466	–	–	–	–	1.7	0.000437	–
217	GLUL	P15104	–	–	0.55	0.000471	Glutamine synthesis
223	SUCLA2	Q9P2R7	–	–	0.55	0.000493	TCA cycle
299	DDAH1	O94760	–	–	0.71	0.000548	NO regulation
227	CKB	P12277	–	–	0.77	0.000996	ATP homeostasis
601	SOD1	P00441	–	–	0.63	0.00102	Antioxidant
419	PAFAH1B2	P68402	–	–	1.5	0.00111	
1028	–	–	–	–	1.5	0.00129	–
443	–	–	–	–	1.9	0.00185	–
530	PRDX1	Q06830	–	–	1.5	0.00214	Antioxidant
653	PRDX5	P30044	–	–	0.77	0.00237	Antioxidant
845	GLUL	P15104	–	–	0.63	0.00238	Glutamine synthesis
375	–	–	–	–	0.71	0.00257	–

Proteins significantly altered in young GBM relative to young peritumoural controls are listed (ordered by *p* value). Only significant protein changes are listed (*p* values shown are prior to Bonferroni correction with a factor 3). Spot ID provides a unique 2DGE spot identifier and is important because several proteins were identified in multiple spots, for example OXCT1 in spot 119 and spot 120. Proteins marked with an asterisk indicate a spot where a second protein (or occasionally more) is present at a level close to that of the listed protein. Blank protein IDs (for example spot 243) represent where protein identity could not be established. The protein accession numbers (Uniprot), magnitude of protein response and *p* values (ranked according to changes in young GBM) are listed for each altered protein. For comparison, proteins significantly altered in old GBM, relative to old controls are listed. Blank values, for example Spot757 (DPYSL2) in old GBM, indicate that the significant change in this protein in young GBM did not achieve statistical significance in the old cohort (see Supplementary Table 3 for more details and information on fold change and probability levels for proteins that failed to reach the pre-determined significance level (i.e. *p* < 0.003)

Immunoblot analysis was performed on a subset of the tissue samples used for proteomics. Proteins (10 μg) were separated by SDS-PAGE and transferred to nitrocellulose membrane. Primary antibodies were detected using fluorescently-labelled secondary antibodies and were visualized using an Odyssey Imager.

To assess functional protein–protein interactions between the proteins altered in young GBM (*p* ≤ 0.01), altered protein identifiers were uploaded to Ingenuity Pathway Analysis (IPA; http://www.ingenuity.com). Networks were algorithmically generated based on direct relationships (physical interactions and/or associations) between eligible proteins. Networks are scored and ranked according to the inclusion of as many proteins inputted as possible. Network scores are putatively a measure of probability [23]. For comparison purposes, network analysis was also performed on proteins differentially regulated in old GBM versus old peritumoural-control. Network analysis is a powerful tool for identifying potential interactions between altered proteins (hypothesis generation) which can be subsequently explored in functional analyses.

## Results

The median survival in the young GBM cohort was >39 months (3 of the 7 patients are still alive) and this was significantly greater than the median survival of 9 months in the older reference group (*p* < 0.02). Performance status (see Supplementary Table 1) was not significantly different between the young (mean age 36 years) and old (mean age 67 years) GBM cohorts.

### Young GBM: proteins differentially expressed in young GBM compared to age matched controls

**Fig. 1:**
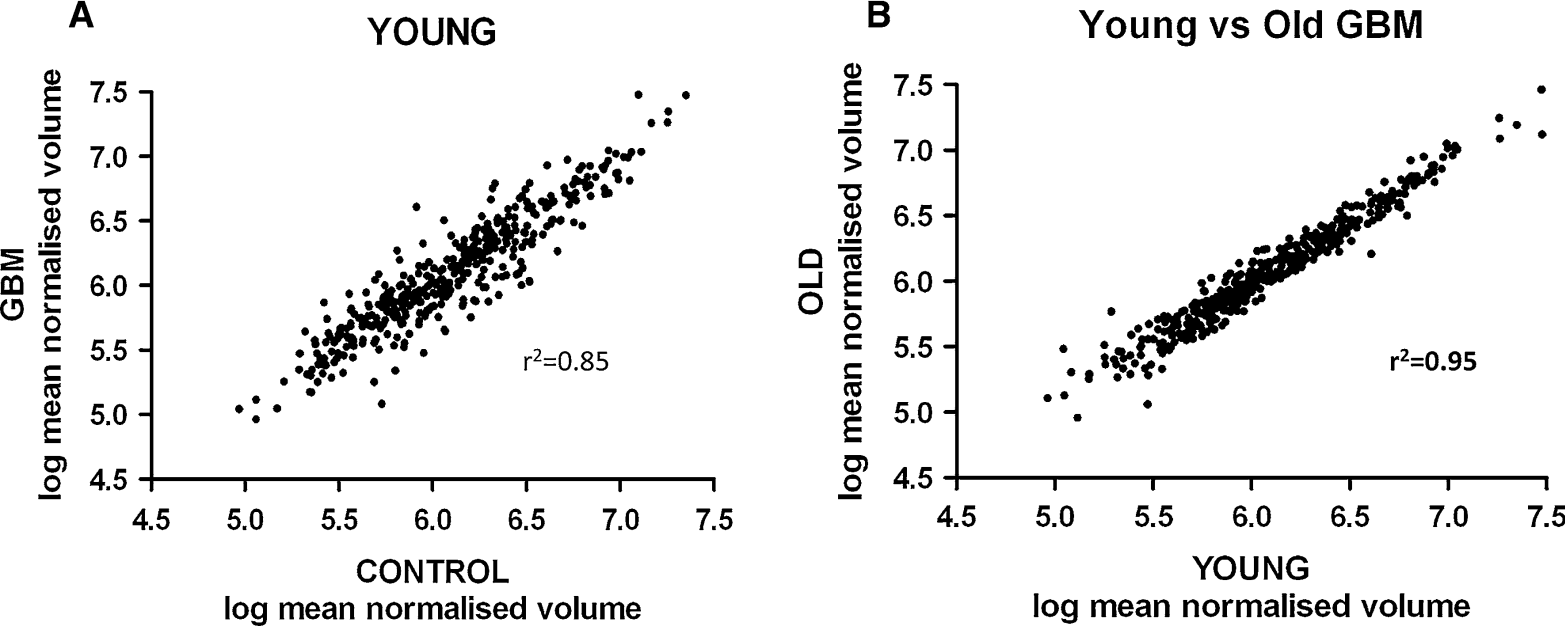
**a** Overview of proteomic analysis of young GBM. Over 400 spots were identified by 2D gel electrophoresis. The normalised volume represents the relative amount of protein in the spot. Each point in the graph represents the relative amount of protein in the 400 spots analysed. Table 1 lists the proteins which are significantly altered in young GBM. In contrast this graph emphasises that the levels of the majority (more than 75 %) of proteins are unaltered in young GBM. Because of the dynamic range (300 fold difference from the most abundant to the least abundant protein), data are presented as logs. There is a good correlation between young GBM and young peritumoural control (r^2^ = 0.85), with 22 % of the spots significantly altered (see Supplementary Table 1). **b** Overview of proteomic response in young GBM compared to old GBM. Over 400 spots were identified by 2D gel electrophoresis. The normalised volume represents the relative amount of protein in the spot. Each point in the *graph* represents the relative amount of protein in the 400 spots analysed. There is an excellent correlation between young GBM and old GBM (r^2^ = 0.95) with only 1 % of the spots significantly altered (5 out of 405; see text for details)

A total of 405 protein spots were matched across every 2Dgel (young GBM and young peritumoural-control gels) and analysed. Logarithmic association of the 405 protein expression levels (mean normalised volumes) highlights multiple protein alterations in young GBM (Fig. 1a, Supplementary Fig. 2). 90 protein spots were altered in young GBM (versus young peritumoural-control; *p* ≤ 0.01) and the identity of these 90 statistically significant altered spots was established by LCMS.

Sixty eight unique proteins were significantly altered in young GBM (Table 1, Supplementary Table 3). 15 of these proteins were identified multiple times in 2–5 spots (ATP6V1B2, OXCT1, ALDOA, GFAP, DCD, DPYSL2, TUBB2A, INA, MBP, ACOT7, VDAC2, UCHL1, PGAM1, PRDX3 and GNB1). Identification of the same protein in several spots is a feature of 2DGE proteomic studies and explains the difference between the number of altered protein spots and number of unique proteins identified. From the 68 altered proteins identified, 29 proteins were up-regulated and 39 proteins were down-regulated. A major fraction of the proteins altered in young GBM (25 %; 16 out of the 68 proteins) are putatively localised to mitochondria (OXCT1, PEBP1, DPYSL2, CKMT1A, ACOT7, CKB, IDH3A, SNAP, VDAC2, PRDX3, HSPD1, CAT, ATP6V1E1, GLUD1, CLIC4 and NDUFS3). 12 of the 68 proteins altered in young GBM have previously been described altered in proteomic studies of glioma (APOA1, GFAP, HSPA5, PDIA3, TUBB2A, GLUD1, GSTP1, PGAM1, UCHL1, HSPB1, HSPD1 and SRI) [5]. Notably, over 50 proteins have been described altered in GBM for the first time.

**Fig. 2:**
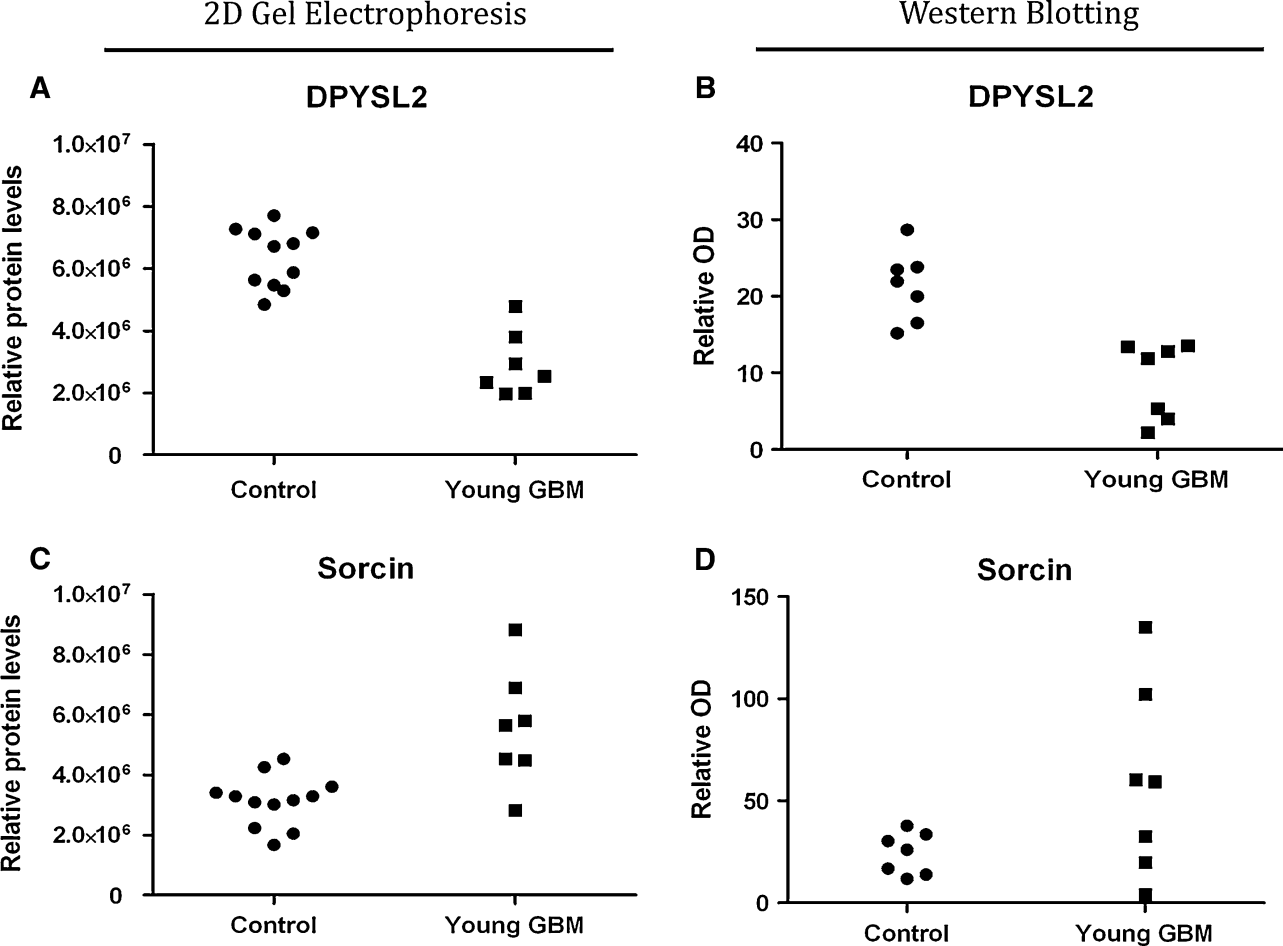
Proteomic alterations in young GBM: confirmation with western analysis. Western blotting replicates the alterations in defined proteins in GBM in a subset (determined by tissue availability) from the same subjects as used in the proteomic 2D gel electrophoresis. **a** 2D gel electrophoresis identified a consistent significant (*p* = 1.8E−06) reduction in DPYSL2 in young GBM. **b** Western blot analysis identified a similar consistent reduction in DPYSL2 in young GBM. **c** 2D gel electrophoresis identified a significant increase (*p* = 0.0057) in Sorcin (though with inter-subject variability) in young GBM. **d** Western blot analysis identified a similar increase in Sorcin in young GBM, again with inter-subject variability. There was also good correspondence between 2D gel electrophoresis and western blot analysis in young GBM for all 10 proteins examined with both techniques (see Supplementary Fig. 3)

Ten proteins (DPYSL2, SRI, OXCT1, UCHL1, CAT, SEPT11, IDH3A, PDIA3, ATP6V1B2, PRDX3), altered in young GBM were examined using western blotting. Western blotting of young GBM versus young peritumoural-control tissue, demonstrated that 7 out of the 10 proteins tested were significantly altered (*p* ≤ 0.01) and that 10 out of the 10 proteins showed the same direction of response as the proteomic analysis (Fig. 2, Supplementary Fig. 3).

IPA network analysis was performed on the proteomic dataset referred to as young GBM and included 68 proteins. The young GBM dataset generated multiple functional protein networks (Table 2) including 4 high scoring networks containing 23, 15, 12 and 11 dataset proteins respectively (Table 2).

**Table 2:**
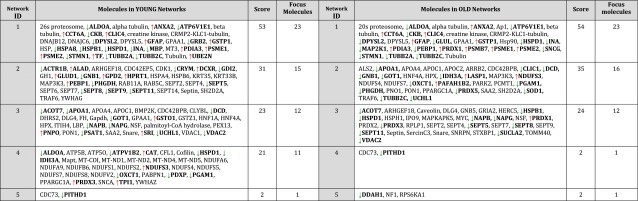
Putative interactions between proteins altered in young GBM and old GBM

Protein–protein interaction networks were generated by IPA (http://www.ingenuity.com). The proteins highlighted in bold are the proteins found significantly altered in the study (*t* test *p* ≤ 0.01, with Bonferroni correction factor 3) in young GBM (relative to young peritumoural control) and old GBM (relative to old control), and are termed ‘Focus Molecules’. Proteins not in bold have been inserted by IPA and are proteins that interact with the focus molecules. The coloured arrows indicate the direction of response of the focus molecules in GBM (red = upregulated; green = downregulated). Each network is assigned a score by IPA. Network scores are putatively a measure of probability for the network (but see [23] for critical analysis of this issue)

The young and old networks display many common features. For example, Network 1 (the highest scoring network) in young GBM contains 23 focus molecules and 17 of these (ALDOA, ANXA2, ATP6V1E1, CCT6A, CKB, CLIC4, DPYSL2, GFAP, GSTP1, HSPD1, INA, PDIA3, PSME1, PSME2, STMN1, TUBB2A, TUBB2C) are also found in Network 1 in old GBM

**Fig. 3:**
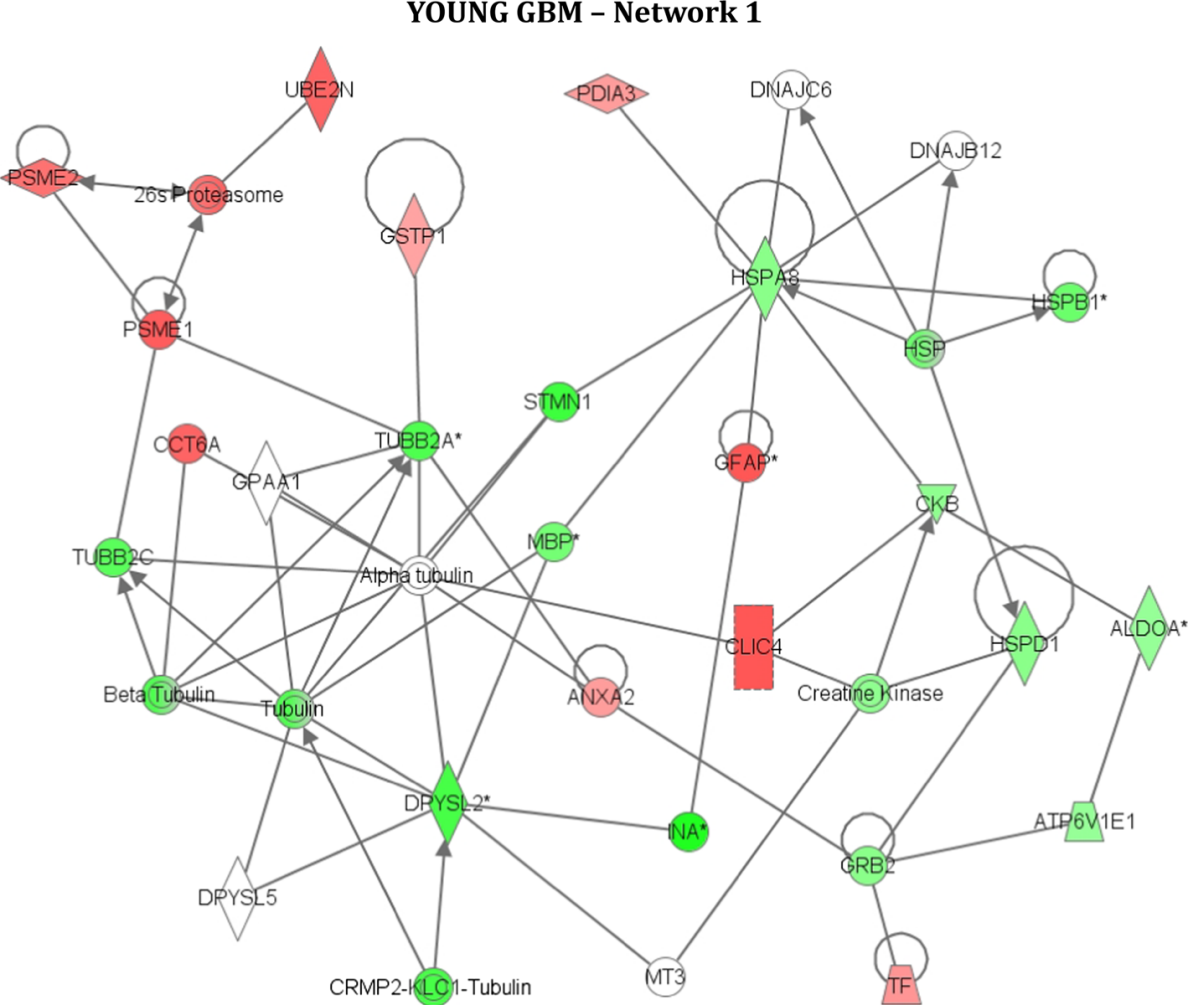
Protein-protein interactions in young GBM. Visual representation of putative protein–protein interactions in Network 1 (the highest scoring network; Table 2) generated by IPA in young GBM. Each node (*shape*) represents a protein and its association with other proteins, is represented by a *line*. Nodes have different shapes that represent different molecule types, for example, transcription factors, enzymes, kinases and phosphatases (refer to Ingenuity Systems Software for detailed node information). Proteins or ‘nodes’ with a coloured background were regulated in the study (*green* = downregulated; *red* = upregulated) whilst other interacting proteins with no background are proteins not detected in this study that have been inserted by IPA to produce a highly connected network. The *solid lines* represent direct interactions or associations between proteins

The top network generated by IPA (Fig. 3) included multiple structural proteins downregulated in young GBM, for example Strathmin (STMN1) and dihydropyrimidinase-related protein 2 (DPYSL2). The network also contained GFAP, upregulated in young GBM, which has long been considered a fundamental and diagnostic feature of glioma [23]. The network included heat shock proteins (HSPD1, HSPA8, HSPB1), and a group of downregulated proteins involved in ATP homeostasis and energy metabolism (ALDOA, ATP6V1E1, CKB, CKMT1A), consistent with existing evidence but also identifying for the first time specific protein networks that may be involved in the dysregulation of energy metabolism in malignant glioma [24]. Lastly a cluster of upregulated proteins, integral to the immunoproteosome (PSME1, PSME2, 20 s/26 s proteosome, PSMB7), was highlighted in the top network. Network 2 was characterised by a cluster of Septin proteins (GTPase proteins that have been shown to play a role in gliomagenesis [25], and the insertion of a hub protein, TRAF6, a signal transducer in NFkappaB signalling. Network 3 was characterised by the insertion of a hub protein HNF4alpha, a transcription factor recently shown to play a role in other neoplasias [26] and Network 4 was characterised by numerous mitochondrial-localised proteins (CAT, IDH3A, NDUFS3 and other complex 1 proteins, OXCT1 and PRDX3).

### Old GBM: proteins differentially expressed in Old GBM compared to age matched control tissue

To allow the extensive protein alterations in young GBM to be compared with those in old GBM, a proteomic evaluation was conducted contemporaneously in old GBM (patients >60 years) using the same technology. A total of 405 protein spots were matched across every 2D gel (old GBM and old peritumoural-control gels) and analysed. Logarithmic association of the 405 protein expression levels (mean normalised volumes) was broadly similar to that seen in young GBM (Supplementary Fig. 2). 70 protein spots were altered in old GBM versus old peritumoural-control (*p* ≤ 0.01).

55 unique proteins were altered significantly in old GBM (listed in Table 1 and Supplementary Table 3 in full). 8 of these proteins were identified multiple times in 2-4 spots (GNB1, INA, ALDOA, SEPT11, HSPB1, CKB, CLIC and GLUL). From the 55 altered proteins identified, 16 proteins were up-regulated and 39 proteins were down-regulated. 19 of the 55 proteins have been reported to be putatively localised to mitochondria (OXCT1, ATP6V1E1, NAPG, NDUFS3, ACOT7, CKB, CKMT1A, DPYSL2, GLUL, HSPD1, IDH3A, PRDX1, PRDX3, PRDX5, PEBP1, SUCLA2, SOD1, UQCRFSL1 and VDAC2; 13 of these 19 proteins were also altered in young GBM). 9 of the 55 proteins have previously been described altered in proteomic studies of glioma (APOA1, GFAP, HSPA5, PDIA3, GSTP1, UCHL1, HSPB1, HSPD1 and PRDX1; 8 of these 9 proteins were also altered in young GBM). Over 40 proteins have been described altered in GBM for the first time (24 of which were also altered in young GBM).

As in young GBM, protein alterations were confirmed by Western blot analysis: 8 out of 10 proteins tested were significantly altered (*p* ≤ 0.01) and 10 out of the 10 proteins showed the same direction of response as the proteomic analysis (Supplementary Fig. 4).

The highest scoring networks for old GBM highlighted considerable commonality to the proteomic response in young GBM (Table 2).

### Proteomic response in young GBM: a comparison with the proteomic response in old GBM

Five protein spots were differentially altered between young and old GBM (*p* ≤ 0.01). Only three unique statistically altered proteins were identified (PEBP1, NDUFA10 and PGK1). The proteins in two spots were not identified. This number of altered proteins lies beneath the multiple testing threshold of potential false positive results in the study. There was an excellent correlation (r^2^ = 0.95) between the level of 405 proteins analysed in GBM from younger patients and their level in GBM from older patients (Fig. 1b). This correlation was similar to that seen in peritumoural-control samples from the two age groups (Supplementary Fig. 2). There were good correlations (r^2^ = 0.85 and 0.90) between the level of 405 proteins in young GBM relative to young peritumoural-controls (Fig. 1a) and old GBM relative to old peritumoural-control (Supplementary Fig. 2) respectively. 48 unique proteins altered in GBM were common in young and old cohorts. The direction and fold change of all 48 proteins was consistent in both young and old GBM. From the top 25 altered protein spots identified in Young GBM (ranked by *p* value), 17 were demonstrated altered in old GBM (*p* ≤ 0.01).

## Discussion

The present study provides a powerful example of how proteomics can reliably test a hypothesis (i.e. is the most important prognostic variable in GBM, age, associated with a distinct response) and demonstrates that proteomics can play an important role in understanding GBM pathophysiology. Definition of the proteomic response in samples from patients with a homogeneous and clinically defined age range (18–45 years) addresses one of the design weaknesses in proteomic studies of GBM to date [5].

Young (<45 years of age) and old (>60 years of age) GBM cohorts with a mean age difference of 31 years and a significantly better median survival despite optimal therapy in the younger cohort were recruited. Multiple protein alterations were detected in young and old GBM versus age matched control tissue, and included a mixture of previously well-characterised protein alterations in GBM (for example, GFAP and UCHL1), and the identification of many ‘highly expected’ heat shock proteins (HSPD1, HSPB1, HSPA5, HSPA8) and cytoskeletal proteins (TUBB2A, TUBB2C), which confirm the robustness of our proteomic data.

One cluster of upregulated proteins (Fig. 3) in both young and old GBM comprised PSME1, PSME2, 20 s/26 s proteosome and PSMB7. These interacting proteins are central to the immunoproteosome (i-proteosome). All proteasomes contain a 20 s subunit flanked by either 19 s subunits or 11 s subunits. In the standard proteasome two 19 s subunits enclose a 20 s subunit of 2α rings sandwiching 2β rings with proteolytic subunits (β1, β2, β5). In the i-proteasome these catalytic subunits are substituted by LMP2, MECL, LMP7 and the 20 s is flanked by two 11 s/PA28 subunits. The 11 s contains 3α & 3β alternating subunits regulated by PSME1 and PSME2 respectively [27], two of the proteins upregulated in our young and old GBM analyses. Inhibition of the 20 s/26 s proteasome by drugs, such as carfilzomib leads to a build up of poly-ubiquinated proteins causing cell cycle arrest, apoptosis and inhibition of tumour growth [28].

i-proteasome function is to provide peptides for MHC-class1-antigen presentation. Interferon increases i-proteasome numbers during inflammation and oxidative damage [29]. 26 s proteasomes are ineffective at degrading oxidised proteins, but i-proteasomes can efficiently process these damaged proteins [27]. Increased PSME1 and PSME2 as a result of interferon, would prevent protein build up and apoptosis. Conversely loss of i-proteasome function, through inhibition of 11 s subunit formation or joining of the 11 s subunit to the 20 s, would have the two fold effect of damaged protein aggregation, leading to apoptosis; and the removal of ‘self’ peptides from the cell surface, alerting the immune system to the malignant tumour cells. Elucidating the mechanisms of GBM immune resistance and causes of immunosuppression is currently an area of intense research and therapeutic effort in GBM [30, 31].

The proteomic analyses of young and old GBM also highlighted multiple proteins (PRDX3, UCHL1, PEBP1, DPYSL2, UBE2 N, GSTO) involved in nuclear factor kappaB (NFkB) regulation. NFkB is a transcription factor capable of mediating many cellular responses and modulates oncogenesis, tumour progression and chemotherapy resistance [32–35]. In the cytoplasm, NFkB is a small protein complex containing two subunits that bind inhibitory kappa B (IkB). IkB binding prevents NFkB translocation to the nucleus. Activation of NFkB, with subsequent translocation to the nucleus can occur through the canonical (utilising IkB kinase, IKK), the non-canonical or the alternative pathway. In the nucleus NFkB regulates transcription of proteins that down-regulate apoptosis, increase cell invasiveness, increase angiogenesis and increase vascular permeability, thereby promoting tumourgenesis [32, 36]. Proteins that regulate NFkB function were altered in GBM. GSTO1 (upregulated in the young GBM analysis) increases IL1β levels which activates IKK. GSTO1 also increases Akt phosphorylation in cells exposed to the pro-apoptotic drug cisplantin. Phosphorylated Akt inhibits apoptosis via NFkB [37]. UBE2 N (also upregulated in the young GBM analysis) is also vital for the activation of IKK via TRAF6 [38]. TRAF6, a core signal transducer in the NFkB pathway was highlighted as a hub protein in IPA Network 2 of both young and old GBM IPA analyses. PRDX3 (downregulated in young and old GBM analyses) also increases IKK activation [39], and knock-down studies of UCHL1 (also downregulated in young and old GBM analyses), show an increase in NFkB function via IKK activation [40]. PEBP1 (upregulated in young GBM and downregulated in old GBM) antagonises NFkB function by interfering with the TNFα pathway [41], resulting in an increase in NFkB function. NFkB’s role in gliomagenesis is summarised in Supplementary Fig. 5. NFkB inhibitors have shown promise in inducing cell death in GBM [42].

Alignment of protein alterations identified in young and old GBM versus age-matched peritumoural-controls showed considerable commonality in the proteomic response of GBM in different aged patients (and also demonstrated the rigour of our two distinct proteomic analyses of GBM). Our study does not provide a clear explanation as to why young and old patients with GBM have differential prognoses. One of the few proteins putatively altered in expression level between young and old GBM, is Phosphatidyl ethanolamine binding protein 1 (PEBP1; also known as Raf1-kinase inhibitor protein, RKIP). PEBP1 was found significantly upregulated in our young GBM proteomic analysis, significantly downregulated in our old GBM analysis, and significantly downregulated in old GBM compared to young GBM. PEBP1 inhibits the RAF/MEK/ERK pro-oncogenic pathway and also inhibits NFkB (also pro-oncogenic) by antagonising the activity of IKK either directly or via Tumour Necrosis Factor alpha (TNFalpha) [41]. The difference in PEBP1 expression levels between young and old GBM could contribute to their different prognosis.

## Electronic supplementary material

Below is the link to the electronic supplementary material.

Supplementary material 1 (DOC 42 kb)

Supplementary material Supplementary Figure 1: Co-efficient of variation in young and old GBM and control. The mean percentage of coefficient of variation of 2D gel electrophoresis analysis is similar across all four of the experimental groups (young control 27.34 %; young GBM 33.55 %; old control 26.39 %; and old GBM 33.99 %) (PPT 123 kb)

Supplementary material Supplementary Figure 2: Overview of Proteomic analysis. Over 400 spots were identified by 2D gel electrophoresis in each cohort. The normalised volume represents the relative amount of protein in the spot. Each point in each graph represents the relative amount of protein in the 400 spots analysed. All data is log transformed. Log transform provides an excellent visual representation of protein variance within the dataset, highlighting protein changes. [A] Proteins expressed in young control *versus* old peritumoural control. There is an excellent correlation between young peritumoural control and old peritumoural control (r^2^=0.97) with little deviation (ie. very small variance) from the line of identity. [B] Proteins expressed in young GBM *versus* young peritumoural control. There is a good correlation between young GBM and young peritumoural control (r^2^=0.85), with 22% of the spots significantly altered (see Supplementary Table 1). [C] Proteins expressed in old GBM *versus* old peritumoural control. There is a good correlation between old GBM and old peritumoural control (r^2^=0.90), with 17% of the spots significantly altered (see Supplementary Table 1). [D] Proteins expressed in young GBM *versus* old GBM. There is an excellent correlation between young GBM and old GBM (r^2^=0.95) with only 1% of the spots significantly altered (5 out of 405; see text for details). (PPT 120 kb)

Supplementary material Supplementary Figure 3: Proteomic alterations in young GBM: Confirmation with western analysis. Western blotting replicates the alterations in defined proteins in young GBM in a subset (determined by tissue availability) from the same subjects as used in the proteomic 2D gel electrophoresis. There was good correspondence in the pattern of response of all proteins examined (OXCT1, UCHL1, Catalase, Septin11, IDH3A, PDIA3, atp6v1b2, PRDX3) in young GBM with 2D gel electrophoresis and western blot analysis. (PPT 642 kb)

Supplementary material Supplementary Figure 4: Proteomic alterations in old GBM: Confirmation with western analysis. Western blotting replicates the alterations in defined proteins in old GBM in a subset (determined by tissue availability) from the same subjects as used in the proteomic 2D gel electrophoresis. There was good correspondence in the pattern of response of all proteins examined (OXCT1, UCHL1, Catalase, Septin11, IDH3A, PDIA3, atp6v1b2, PRDX3) in old GBM with 2D gel electrophoresis and western blot analysis. (PPT 629 kb)

Supplementary material Supplementary Figure 5: Nuclear Factor kappa B signaling in young and old GBM. Several proteins found altered in young and old GBM (PRDX3, UCHL1, PEBP1, DPYSL2, UBE2N and GSTO) are known to play a role in Nuclear Factor kappa B (NFkB) signalling. This schematic summarises the putative links to NFkB signalling in young and old GBM and the potential roles of NFkB in gliomagenesis. Modulation of NFkB function is frequently via IKK (= Inhibitory kappa B). Proteins marked in red were upregulated in GBM and proteins marked in green were downregulated in GBM in the proteomic study. Proteins marked in Blue were altered in GBM but differentially altered in different protein spots. (PPT 330 kb)

Supplementary material 7 (DOCX 17 kb)

Supplementary material 8 (DOC 54 kb)

Supplementary material 9 (DOC 154 kb)
