# Supplementary material for: The proteomic response in glioblastoma in young patients
Source: J Neurooncol. 2014 May 18;119(1):79–89. doi: 10.1007/s11060-014-1474-6 (PMC4129242; doi:10.1007/s11060-014-1474-6)
Supplement: Supplementary file 1 — Supplementary material 1 (DOC 42 kb) [file 11060_2014_1474_MOESM1_ESM.doc]

**MATERIALS AND METHODS**

**Clinical Material**

Glioblastoma and peritumoural control brain samples were obtained from young (<45yrs) and old patients (>60yrs) undergoing resective brain tumour surgery after informed written consent (Lothian Region Ethical Approval LREC/2004/4/16). Glioblastoma samples were removed from viable tumour tissue identified intraoperatively using operating loupes and Intraoperative image guidance (BrainLAB, Munich) by a single neurosurgeon. In the operating theatre a small sample of each specimen was also taken for histological analysis to confirm the tumour grading (WHO) and sample characteristics. Peritumoural ‘control’ tissue was also determined using a BrainLAB MRI guided system (merged T1 contrast enhanced plus T2) and similarly harvested. The clinical details (patient age, assessment of performance status, patient gender, patient pathology, medications and prior therapies) of every tissue sample used for proteomic analysis can be found in Supplementary Tables 1 and 2. Immediately following removal in theatre both GBM and peritumoural control brain were homogenised on ice using a tight fitting Dounce glass homogeniser in 9M urea:4%CHAPS buffer supplemented with protease inhibitors (Complete: Roche). Samples were then centrifuged at 14,000xg for 20minutes, the soluble component decanted, and the total protein yield quantified using the BCA protein assay (Perbio Science, UK).

The experimental group sizes used for the primary proteomic analysis were as follows: young GBM (n=7) and young peritumoural control (n=12) (based on *a priori* power calculations to detect significant changes of ≥35% with power≥0.8). Tissue was also collected for two comparison groups: old GBM (n=13) and old peritumoural control (n=10). Note the median co-efficients of variation were similar in each experimental group: young GBM 33.55%, young peritumoural control 27.38%, old GBM 33.99% and old peritumoural control 26.39% (see Supplementary Fig.1).

**Two dimensional gel electrophoresis (2DGE)**

Total protein extracts were separated in two dimensions using 2DGE. 2DGE separates proteins according to their *pI* (isoelectric point) (using 24cm nonlinear pH3-10 IPG strips) and their *Mr* (molecular mass). Separation in the first dimension (*pI*) was performed using 24cm nonlinear pH3-10 IPG strips (GE Healthcare) using a BioRad protean IEF cell. Samples were diluted in IEF sample buffer (7M Urea, 2M thiourea, 4% CHAPS, 1%DTT, 1% ampholytes) to reach a final protein load of 400μg (in 410μl) per strip. IPG strips were actively rehydrated for 12 hours (50V) and then focussed as follows: 500V for 1hr, 1000V for 1 hr and 8000V for 32000Vhrs. DTT (3.5%) was added to the cathode to maintain reducing conditions. Once focussed, IPG strips were equilibrated twice for 15minutes in equilibration buffer (0.1M tris-Hcl pH6.8, 5.5M urea, 0.3% Glycerol and 0.035% SDS) containing 1% DTT for the first equilibration and 2.5% iodoacetamide for the second equilibration. Separation in the second dimension (*Mr*) was performed using 12.5% SDS-polyacrylamide gels (24cm) at 1 Watt *per* gel for 18 h using the manufacturer’s proprietary buffer system.

Gels were fixed for at least 30minutes in 40% methanol:7% acetic acid and stained for 3hours with Sypro Ruby. Gels were destained with 40% methanol:7% acetic acid for a minimum of 30 minutes prior to imaging and were then stored at 4^o^C in deionized water.

**2DGE image analysis**

2D gel images were captured using a FluorChem Image Analyser. Duplicate gels were run for each sample but only the best gel was kept for further analysis with Progenesis Samespots software. Gel images were analysed to identify changes in protein levels between three comparisons of interest: young GBM *versus* young peritumoural control, old GBM *versus* old peritumoural control and young GBM *versus* old GBM. Gel images (from the two primary experimental groups young GBM and young peritumoural control plus the two comparison groups old GBM and old peritumoural control; n=42) were aligned in a single study using at least four manual alignment vectors placed on key landmark features on each gel followed by automatic placement of further alignment vectors by the software (typically 200 – 400 vectors per gel). On average, 700 spots relating to proteins with a pI between 3 and 10 and a molecular weight from 10 to 250kDa were detected on each analytical gel and 405 protein spots were matched across every gel in the study. Gels were then grouped according to sample pathology and age (see Supplementary Table 1 and Supplementary Table 2). The relative protein levels were assessed by the calculation of densities of protein features normalised to the total protein detected on the gel. Finally, the mean protein levels of each protein were analysed using Student’s t-test (p≤0.003, equivalent to p≤0.01 with Bonferroni correction factor 3 for each comparison of interest described above). Significant data are presented in Table 1 (uncorrected for multiple comparisons) and all data are presented in Supplementary Table 3.

**In gel trypsin digestion**

Protein spots differentially expressed in young GBM *versus* young peritumoural control (p≤0.01 with Bonferroni correction factor 3) were manually excised and washed three times in 50mM ammonium bicarbonate:50% acetonitrile (ACN). Each gel piece was dehydrated in 100% ACN, fully dried, rehydrated with 12.5ug/ml unmodified sequencing grade trypsin and incubated at 30^o^C for 16-24hours. Each peptide mixture was then sonicated for 10minutes, cleaned on ZipTip (Millipore, UK) according to manufacturer’s instructions and dried using a vacuum drier. An identical approach was applied in parallel comparing old GBM *versus* old peritumoural control, and young GBM *versus* old GBM.

**Protein Identification using LC/MS**

Capillary-HPLC-MSMS analysis was carried out using an on-line system consisting of a micro-pump (1200 binary HPLC system) coupled to a hybrid LTQ-Orbitrap XL instrument. The LTQ-Orbitrap XL was controlled through Xcalibur 2.0.7. The HPLC-MS methods have previously been described [14]. Samples were reconstituted in 10 µl loading buffer, injected, and analyzed on a 1 hour gradient for data dependant analysis. Conversions from RAW to MGF files were performed as previously described [14]. MS data were interrogated using MASCOT Version 2.3 (matrix Science Ltd, UK) against a human IPI database downloaded from [www.ebi.ac.uk](http://www.ebi.ac.uk/) version 3.42. Variable methionine oxidation, STY phosphorylation, protein N-terminal acetylation and fixed cysteine carbamidomethylation were used in all searches. Precursor mass tolerance was set to 7 ppm and MSMS tolerance was set to 0.4 mu. The significance threshold (p) was set below 0.05 (MudPIT scoring). Some complementary searches were performed using the “error tolerant” feature of Mascot in order to identify other potential amino acid modifications which were not part of the group mentioned above. LC-MS runs of each sample were combined using Maxquant (version 1.0.13.8), assuming a false positive rate of 0.01 [15]. Proteins identified were ranked based on their maximum intensity as extracted by Maxquant and single-peptide hits were discarded. The highest intensity hit was taken forward as the identified protein.

**Immunoblotting**

Immunoblot analysis was performed on a subset of the tissue samples used for proteomics. Proteins (10μg) were separated by SDS-PAGE and transferred to nitrocellulose membrane. All blots were stained with Ponceau S to ensure equal protein loading across lanes before proceeding. Immunoblotting was performed using the Odyssey Infrared Imaging System (LiCor Biosciences). Membranes were blocked in Odyssey blocking buffer (diluted 1:1 with PBS) and washed in PBS-Tween (PBS with 0.1% Tween). Primary antibodies were detected using fluorescently labelled secondary antibodies (IRDye680 or IRDye800, 1:10,000). Proteins were visualized by scanning antibody labelled blots in the Odyssey Imager under the appropriate channel. Primary antibodies used for western blot analysis were sourced from Abcam with the exception of Sorcin (LSBio), and GAPDH (Sigma). Blots were labelled with either GAPDH or alpha-tubulin antibodies to assess protein loading between samples of similar pathology. To analyse the protein levels, densitometry was performed using the Odyssey infrared image analysis program. Protein bands were selected, background subtraction applied, and a resulting optical density (OD) value computed. Protein OD levels were not normalised to loading controls since GAPDH and alpha-tubulin may be regulated in tumours.

**Network analysis**

To assess whether functional protein–protein interactions existed between the proteins altered in young GBM (p≤0.01), altered protein identifiers were uploaded to Ingenuity Pathway Analysis software (http://www.ingenuity.com). Networks were algorithmically generated based on direct relationships (physical interactions and/or associations) between eligible proteins. The networks are colour coded with green protein nodes representing proteins that were downregulated in GBM and red protein nodes representing proteins that were upregulated in GBM. The shading of network nodes is negatively correlated to the magnitude of fold change. Networks are scored and ranked according to the inclusion of as many proteins that were inputted as possible. Network scores are putatively a measure of probability for the network (but see [16] for critical analysis). For comparison purposes, network analysis was also performed (as above) on proteins differentially regulated in old GBM *versus* old peritumoural control.
