# Supplementary figures and images for: The proteomic response in glioblastoma in young patients
Source: J Neurooncol. 2014 May 18;119(1):79–89. doi: 10.1007/s11060-014-1474-6 (PMC4129242; doi:10.1007/s11060-014-1474-6)

## Slide 1
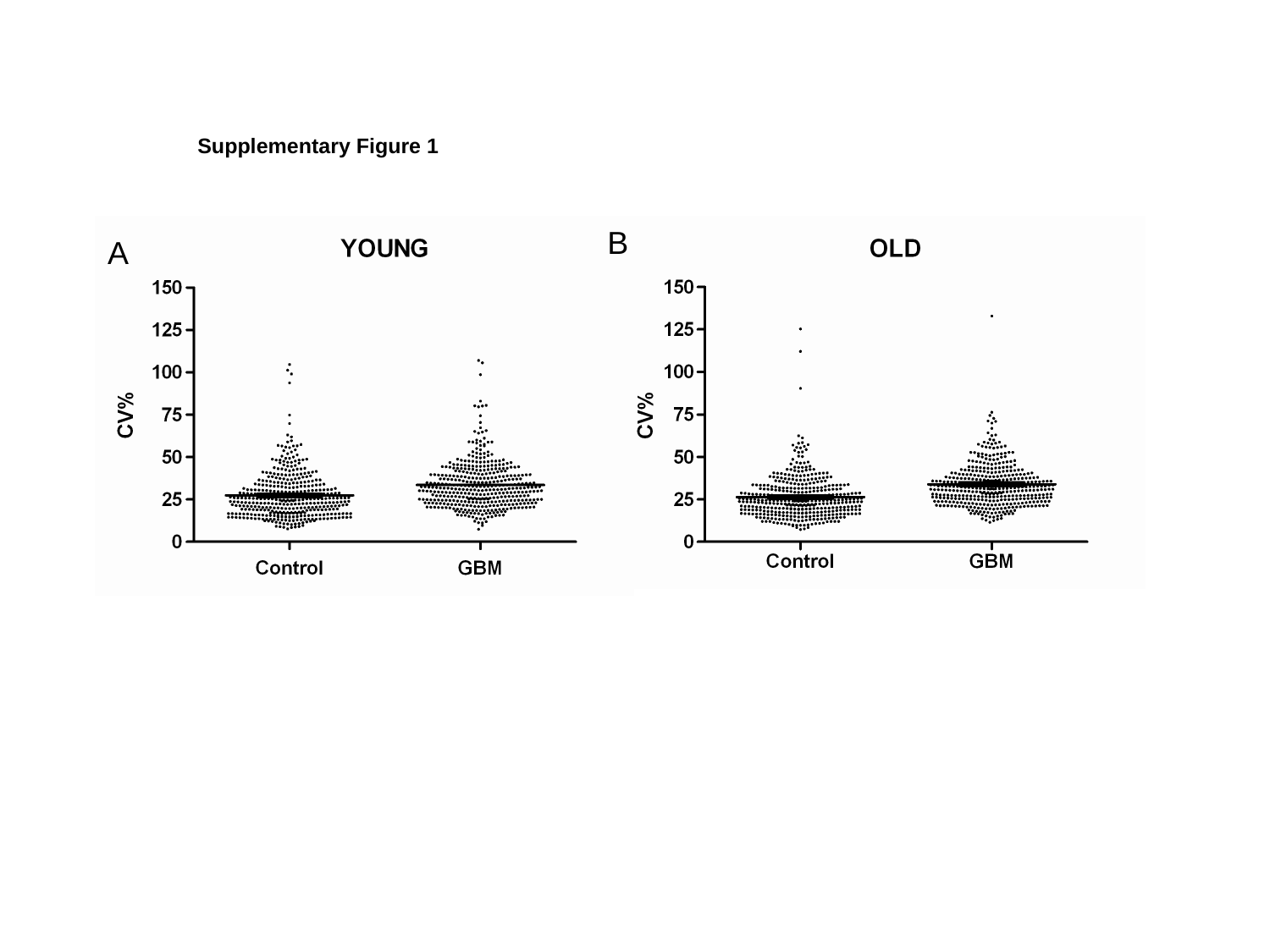

Supplementary Figure 1
B
A

Supplement: Supplementary file 2 — Supplementary material Supplementary Figure 1: Co-efficient of variation in young and old GBM and control. The mean percentage of coefficient of variation of 2D gel electrophoresis analysis is similar across all four of the experimental groups (young control 27.34 %; young GBM 33.55 %; old control 26.39 %; and old GBM 33.99 %) (PPT 123 kb) [file 11060_2014_1474_MOESM2_ESM.ppt]

## Slide 1
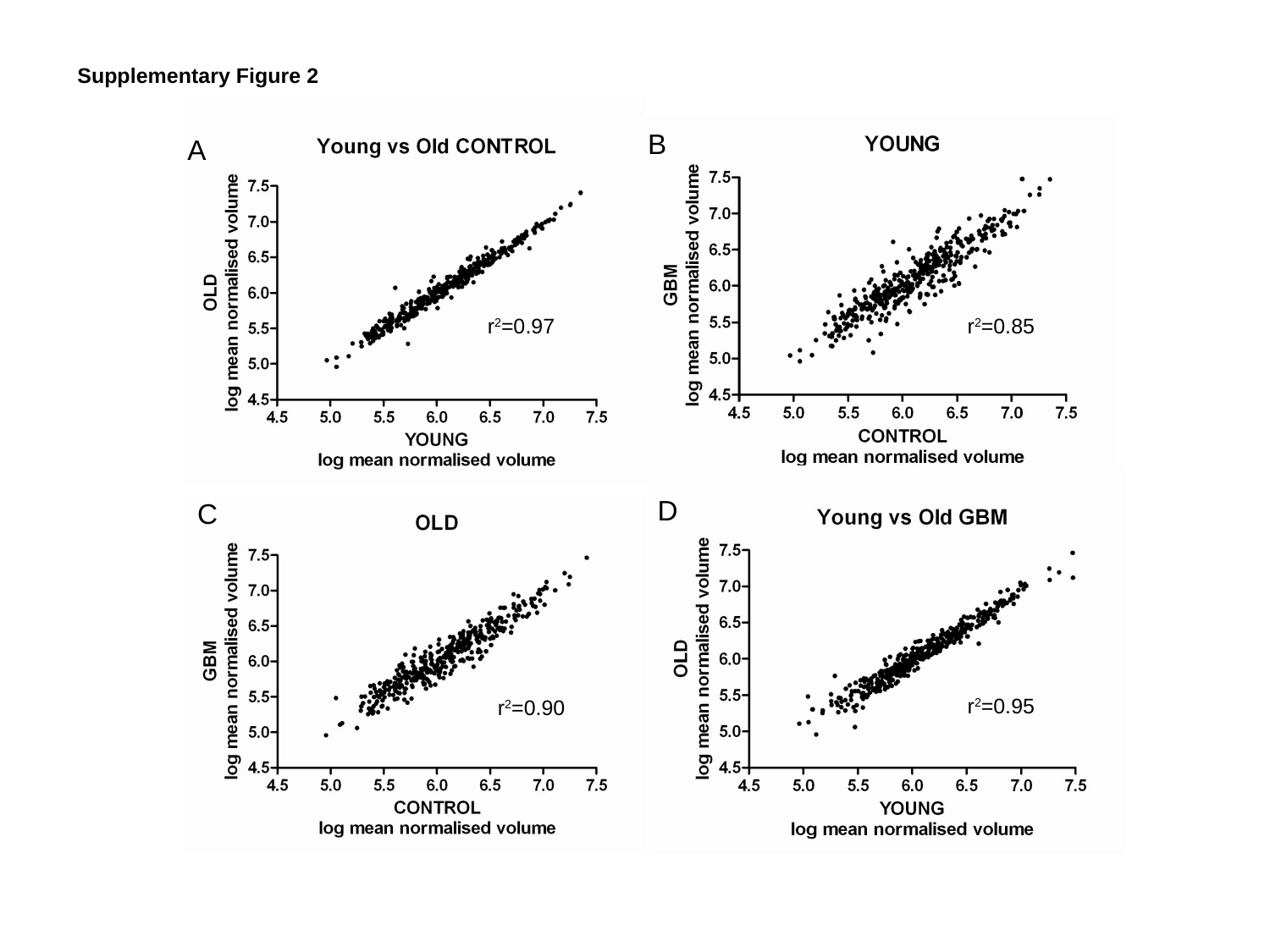

Supplementary Figure 2
B
A
r2=0.97
r2=0.85
D
C
r2=0.95
r2=0.90

Supplement: Supplementary file 3 — Supplementary material Supplementary Figure 2: Overview of Proteomic analysis. Over 400 spots were identified by 2D gel electrophoresis in each cohort. The normalised volume represents the relative amount of protein in the spot. Each point in each graph represents the relative amount of protein in the 400 spots analysed. All data is log transformed. Log transform provides an excellent visual representation of protein variance within the dataset, highlighting protein changes. [A] Proteins expressed in young control versus old peritumoural control. There is an excellent correlation between young peritumoural control and old peritumoural control (r2=0.97) with little deviation (ie. very small variance) from the line of identity. [B] Proteins expressed in young GBM versus young peritumoural control. There is a good correlation between young GBM and young peritumoural control (r2=0.85), with 22% of the spots significantly altered (see Supplementary Table 1). [C] Proteins expressed in old GBM versus old peritumoural control. There is a good correlation between old GBM and old peritumoural control (r2=0.90), with 17% of the spots significantly altered (see Supplementary Table 1). [D] Proteins expressed in young GBM versus old GBM. There is an excellent correlation between young GBM and old GBM (r2=0.95) with only 1% of the spots significantly altered (5 out of 405; see text for details). (PPT 120 kb) [file 11060_2014_1474_MOESM3_ESM.ppt]

## Slide 1
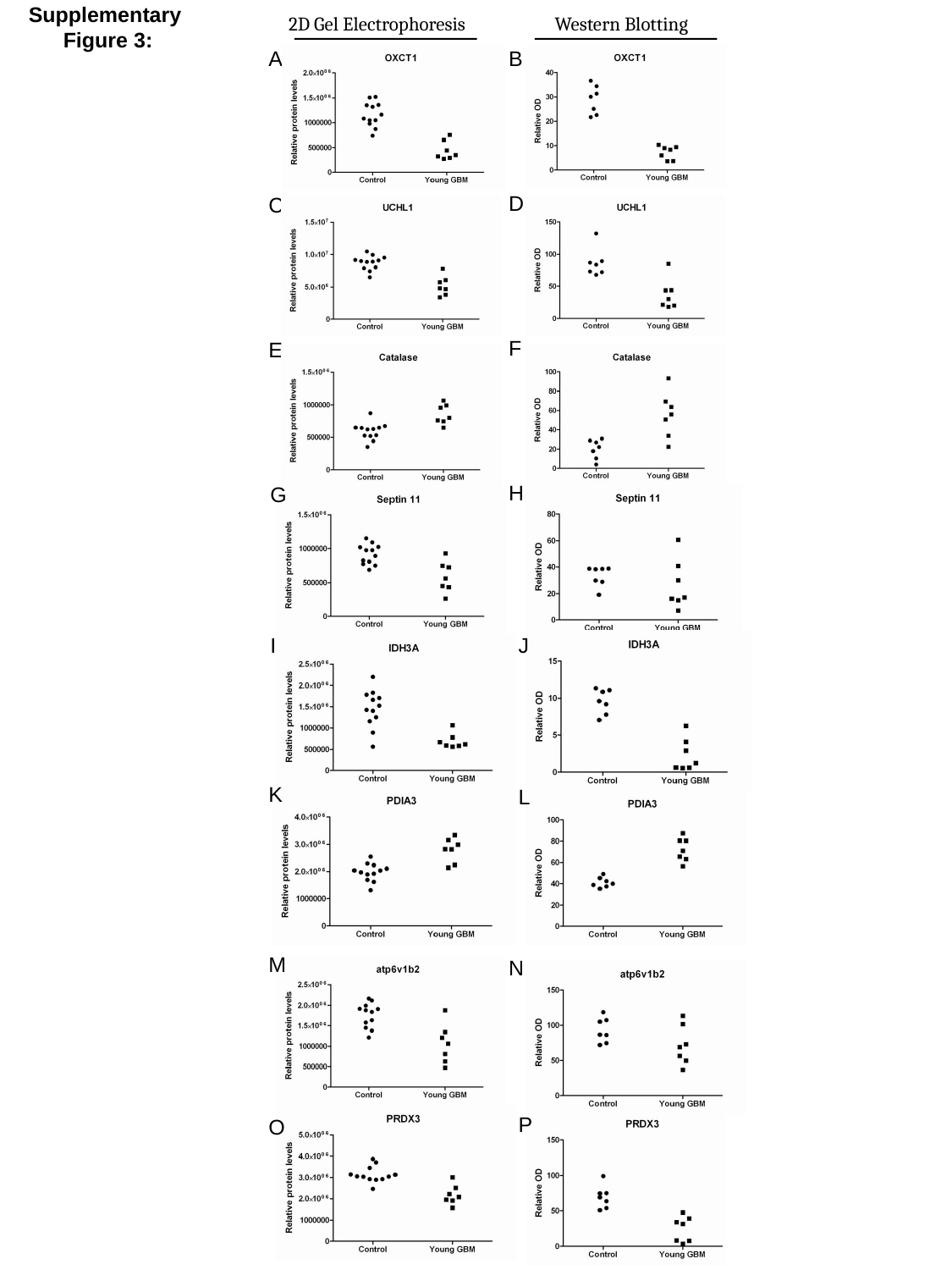

Supplementary
Figure 3:
2D Gel Electrophoresis
Western Blotting
A
B
D
C
F
E
H
G
I
J
K
L
M
N
P
O

Supplement: Supplementary file 4 — Supplementary material Supplementary Figure 3: Proteomic alterations in young GBM: Confirmation with western analysis. Western blotting replicates the alterations in defined proteins in young GBM in a subset (determined by tissue availability) from the same subjects as used in the proteomic 2D gel electrophoresis. There was good correspondence in the pattern of response of all proteins examined (OXCT1, UCHL1, Catalase, Septin11, IDH3A, PDIA3, atp6v1b2, PRDX3) in young GBM with 2D gel electrophoresis and western blot analysis. (PPT 642 kb) [file 11060_2014_1474_MOESM4_ESM.ppt]

## Slide 1
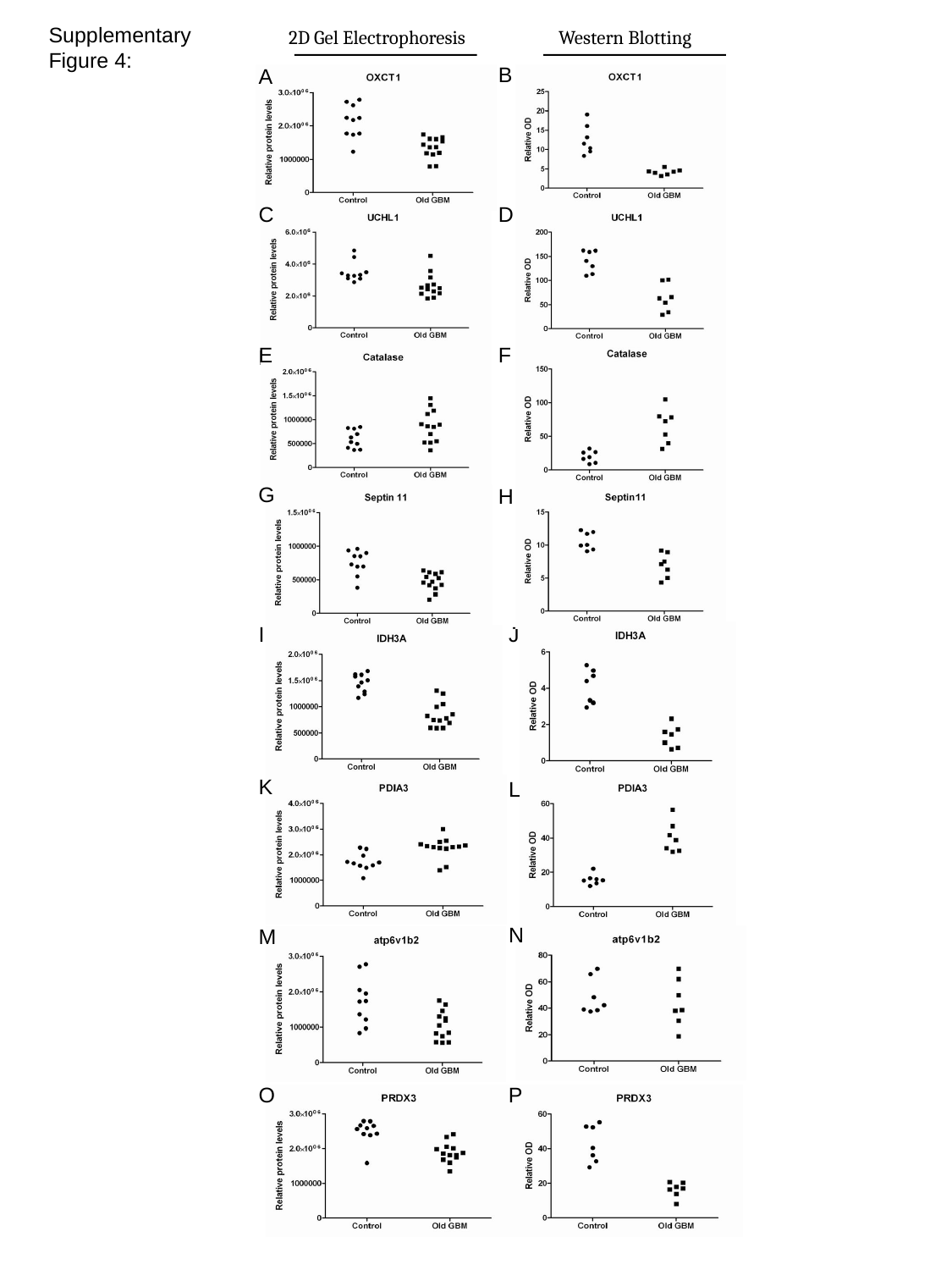

Supplementary Figure 4:
2D Gel Electrophoresis
Western Blotting
B
A
C
D
E
F
E
G
H
I
J
K
L
N
M
O
P

Supplement: Supplementary file 5 — Supplementary material Supplementary Figure 4: Proteomic alterations in old GBM: Confirmation with western analysis. Western blotting replicates the alterations in defined proteins in old GBM in a subset (determined by tissue availability) from the same subjects as used in the proteomic 2D gel electrophoresis. There was good correspondence in the pattern of response of all proteins examined (OXCT1, UCHL1, Catalase, Septin11, IDH3A, PDIA3, atp6v1b2, PRDX3) in old GBM with 2D gel electrophoresis and western blot analysis. (PPT 629 kb) [file 11060_2014_1474_MOESM5_ESM.ppt]

## Slide 1
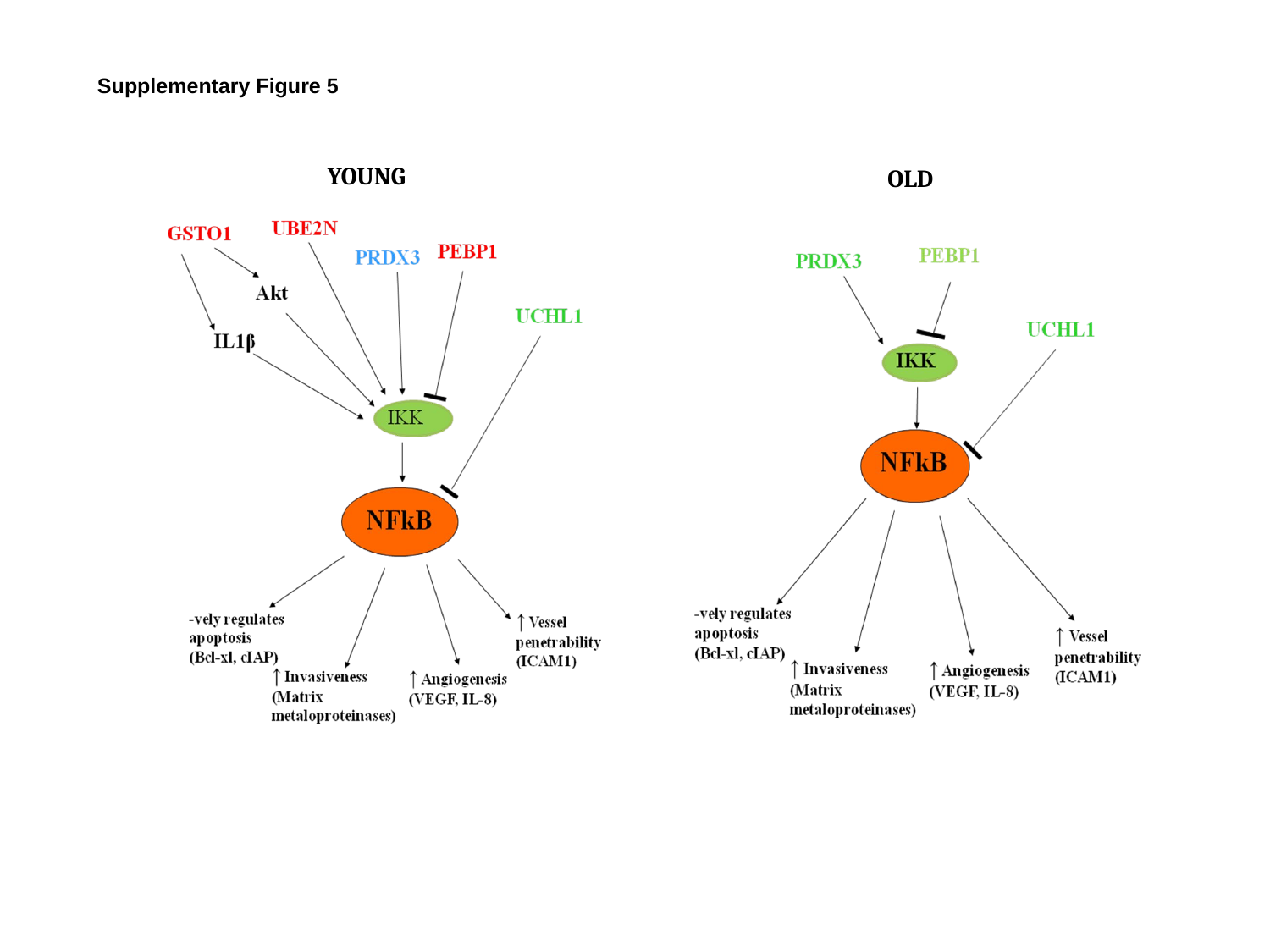

Supplementary Figure 5
YOUNG
OLD

Supplement: Supplementary file 6 — Supplementary material Supplementary Figure 5: Nuclear Factor kappa B signaling in young and old GBM. Several proteins found altered in young and old GBM (PRDX3, UCHL1, PEBP1, DPYSL2, UBE2N and GSTO) are known to play a role in Nuclear Factor kappa B (NFkB) signalling. This schematic summarises the putative links to NFkB signalling in young and old GBM and the potential roles of NFkB in gliomagenesis. Modulation of NFkB function is frequently via IKK (= Inhibitory kappa B). Proteins marked in red were upregulated in GBM and proteins marked in green were downregulated in GBM in the proteomic study. Proteins marked in Blue were altered in GBM but differentially altered in different protein spots. (PPT 330 kb) [file 11060_2014_1474_MOESM6_ESM.ppt]
