# Supplementary material for: The proteomic response in glioblastoma in young patients
Source: J Neurooncol. 2014 May 18;119(1):79–89. doi: 10.1007/s11060-014-1474-6 (PMC4129242; doi:10.1007/s11060-014-1474-6)
Supplement: Supplementary file 7 — Supplementary material 7 (DOCX 17 kb) [file 11060_2014_1474_MOESM7_ESM.docx]

**Supplementary Table 1: Proteomic alterations and clinical details for the young and old GBM cohorts.**

|  | **ID** | **Age** | **Sex** | **Surgery** | **RT** | **RT+TMZ** | **Chemotherapy** | **Survival (months)** | **KPS** | **Protein Spots analysed** | **Protein Spots altered p≤0.01** | **Altered protein  IDs p≤0.01** | **Common  altered protein  IDs p≤0.01** |
| --- | --- | --- | --- | --- | --- | --- | --- | --- | --- | --- | --- | --- | --- |
| **YOUNG GBM**  *mean age = 36yrs*  *age range: 18-45yrs* | 1 | 18 | F | Resection | - | 60G | 6 cycles TMZ | alive, 40+ | 100 | 405 | 90 | 68 | 48 |
|  | 2* | 31 | F | Resect + gliadel | - | - | 4 cycles TMZ | 32 | 90 |  |  |  |  |
|  | 3 | 36 | M | Resection | - | 60G | 6 cycles TMZ | alive, 39+ | 100 |  |  |  |  |
|  | 4 | 38 | M | Resect + gliadel | 60G | - | ? | ? | 90 |  |  |  |  |
|  | 5 | 43 | M | Resection | - | 60G | 1TMZ + 4PCV | 17 | 80 |  |  |  |  |
|  | 6* | 43 | M | Resection | - | 60G | 6 cycles TMZ | alive, 39+ | 90 |  |  |  |  |
|  | 7 | 45 | M | Resection | - | 60G | 6 cycles TMZ | 19 | 90 |  |  |  |  |
| **OLD GBM**  *mean age = 67yrs*  *age range: 60-74yrs* | 20 | 63 | F | Resection | - | 60G | 6 cycles TMZ | 17 | 100 | 405 | 70 | 55 |  |
|  | 21 | 68 | M | Resect + gliadel | 60G | - | 1 cycle TMZ | 8 | 80 |  |  |  |  |
|  | 22 | 72 | M | Resection | 30G in 6# | - | - | 9 | 90 |  |  |  |  |
|  | 23 | 67 | M | Resect + gliadel | 60G | - | - | 20 | 90 |  |  |  |  |
|  | 24 | 61 | F | Resection | 30G in 6# | - | - | 3 | 70 |  |  |  |  |
|  | 25 | 68 | F | Resection | 60G | - | 2 cycles PCV | 9 | 90 |  |  |  |  |
|  | 26 | 63 | F | Resection | - | 60G | 2 cycles TMZ | alive, 22 | 100 |  |  |  |  |
|  | 27 | 60 | M | Resection | - | 60G | - | 4 | 80 |  |  |  |  |
|  | 28 | 72 | M | Resection | - | 60G | 4 cycles | 15 | 90 |  |  |  |  |
|  | 29 | 74 | F | Resection | 30G in 6# | - | 2 cycles PCV | 14 | 80 |  |  |  |  |
|  | 30 | 63 | F | Resection | 30G in 6# | - | - | 4 | 80 |  |  |  |  |
|  | 31 | 69 | F | Resection | - | 60G | 1 cycle TMZ | 6 | 90 |  |  |  |  |
|  | 32 | 69 | M | Resection | 60G | - | - | 9 | 90 |  |  |  |  |

All patients had glioblastoma (GBM) confirmed by a consultant neuropathologist. Resections in all cases were maximal. Younger patients had median survival significantly longer than the older patients (p = 0.02). There was no significant difference in the Karnovsky Performance Status (KPS) between groups. Abbreviations G = gray; # = fractions of radiotherapy; TMZ temozolomide; RT + TMZ = combined chemoradiotherapy; PCV = procarbazine, vincristine, CCNU chemotherapy regime; * = patients diagnosed with secondary glioblastoma. Patient no 4 returned to his homeland after radiotherapy and despite our best efforts was lost to follow up after 3 months.

405 protein spots were identified using 2 Dimensional Gel Electrophoresis in each age cohort. The number of protein spots and identified proteins significantly altered in each GBM age cohort relative to age matched controls is indicated. The overwhelming majority of proteins altered in young and old GBM were common to both age cohorts (i.e. 48 proteins were altered in both young GBM and old GBM). Statistical significance was determined with Students t-test with probability set at p ≤ 0.01 after Bonferroni correction of 3.
