# Supplementary material for: The proteomic response in glioblastoma in young patients
Source: J Neurooncol. 2014 May 18;119(1):79–89. doi: 10.1007/s11060-014-1474-6 (PMC4129242; doi:10.1007/s11060-014-1474-6)
Supplement: Supplementary file 8 — Supplementary material 8 (DOC 54 kb) [file 11060_2014_1474_MOESM8_ESM.doc]

**Supplementary Table 2**

|  | **Patient ID** | **Age** | **Sex** | **Patient pathology** | **Surgery** |
| --- | --- | --- | --- | --- | --- |
| **YOUNG CONTROL**  *mean age = 32yrs*  *age range: 18-43yrs* | 8 | 18 | F | GBM | Resection |
|  | 9 | 21 | F | AII | Resection |
|  | 10 | 23 | F | DNET | Resection |
|  | 11 | 31 | F | MII | Resection |
|  | 12 | 32 | F | 2^o^ GBM | Resection |
|  | 13 | 37 | M | AII | Resection |
|  | 14 | 40 | M | AIII | Resection |
|  | 15 | 41 | M | GBM | Resection |
|  | 16 | 42 | M | AII | Resection |
|  | 17 | 43 | M | GBM | Resection |
|  | 18 | 34 | M | OII | Resection |
|  | 19 | 26 | M | AIII | Resection |
| **OLD CONTROL**  *mean age = 67yrs*  *age range: 60-73yrs* | 33 | 63 | F | GBM | Resection |
|  | 34 | 72 | M | GBM | Resection |
|  | 35 | 63 | F | Melanoma | Resection |
|  | 36 | 66 | F | MI | Resection |
|  | 37 | 73 | F | MI | Resection |
|  | 38 | 68 | F | GBM | Resection |
|  | 39 | 60 | M | GBM | Resection |
|  | 40 | 74 | F | GBM | Resection |
|  | 41 | 69 | F | GBM | Resection |
|  | 42 | 60 | F | ? | Resection |

**Supplementary Table 2: Clinical details for the young and old control cohorts.** Peritumoural ‘control’ tissue was harvested from patients undergoing various types of brain tumour surgery (see ‘patient pathology’). The mean ages of the young (32 years) and old (67 years) control cohorts are similar to the two GBM cohorts described in Supplementary Table 1. Stratification of the experimental groups (for example by IDH1 mutations or methylation status) would be of interest but is precluded by small group sizes relative to the number of proteins assessed (the ‘Curse of dimensionality’).
