# Supplementary material for: The proteomic response in glioblastoma in young patients
Source: J Neurooncol. 2014 May 18;119(1):79–89. doi: 10.1007/s11060-014-1474-6 (PMC4129242; doi:10.1007/s11060-014-1474-6)
Supplement: Supplementary file 9 — Supplementary material 9 (DOC 154 kb) [file 11060_2014_1474_MOESM9_ESM.doc]

**Supplementary Table 3**

| Spot ID | Protein ID | Protein Accession Number | **YOUNG GBM** | | **OLD GBM** | | Main Protein Function |
| --- | --- | --- | --- | --- | --- | --- | --- |
|  |  |  | Fold change | p value | Fold change | p value |  |
| 746 | CKMT1A | P12532 | 0.32 | 1.76E-08 | 0.53 | 3.95E-05 | ATP homeostasis |
| 749 | GNB1***** | P62873 | 0.59 | 2.05E-07 | 0.63 | 2.29E-05 | GPCR beta subunit |
| 757 | DPYSL2 | Q16555 | 0.4 | 3.94E-07 | *0.63* | *0.00759* | Cytoskeletal |
| 798 | INA | Q16352 | 0.36 | 4.21E-07 | 0.59 | 0.00240 | Cytoskeletal |
| 271 | ALDOA | P04075 | 0.71 | 5.48E-07 | 0.77 | 0.00034 | Glycolysis |
| 310 | CRYM | Q14894 | 0.37 | 7.79E-07 | 0.55 | 9.96E-05 | - |
| 768 | STMN1 | P16949 | 0.38 | 8.37E-07 | 0.59 | 0.000146 | Cytoskeletal |
| 161 | GDI2 | P50395 | 0.34 | 9.28E-07 | *0.59* | *0.0341* | - |
| 119 | OXCT1 | P55809 | 0.37 | 9.71E-07 | *0.53* | *0.00423* | Lipid metabolism |
| 67 | DPYSL2 | Q16555 | 0.45 | 1.11E-06 | 0.63 | 0.00246 | Cytoskeletal |
| 760 | VDAC2 | P45880 | 0.53 | 1.28E-06 | 0.53 | 1.40E-06 | Ion transport |
| 763 | GOT1 | P17174 | 0.53 | 2.09E-06 | 0.53 | 1.86E-05 | Amino acid metabolism |
| 736 | GNB1***** | P62873 | 0.43 | 2.32E-06 | 0.47 | 1.05E-06 | GPCR beta subunit |
| 343 | NAPB | Q9H115 | 0.5 | 2.34E-06 | 0.55 | 7.05E-06 | Ca2+ mediated exocytosis |
| 469 | NDUFS3 | O75489 | 2.9 | 2.83E-06 | 2.5 | 3.00E-06 | Electron transport |
| 492 | C1orf128 | Q9GZP4 | 0.36 | 3.55E-06 | 0.5 | 2.36E-05 | unknown |
| 120 | OXCT1 | P55809 | 0.48 | 3.70E-06 | 0.63 | 0.00026 | Lipid metabolism |
| 451 | PNPO***** | B4E152 | 2.1 | 6.70E-06 | *1.4* | *0.0086* | Pyridoxine biosynthesis |
| 249 | TUBB2A | Q13885 | 0.5 | 7.44E-06 | *0.77* | *0.00535* | Cytoskeletal |
| 243 | - | - | 0.59 | 1.25E-05 | 0.59 | 1.29E-05 | - |
| 938 | MBP | P02686 | 0.53 | 1.36E-05 | *0.83* | *0.0614* | Myelin |
| 809 | PSAT1 | Q9Y617 | 0.53 | 1.45E-05 | *0.77* | *0.0116* | Amino acid biosynthesis |
| 84 | INA | Q16352 | 0.33 | 1.51E-05 | 0.45 | 3.21E-05 | Cytoskeletal |
| 613 | PGAM1 | P18669 | 0.33 | 1.68E-05 | 0.55 | 0.00044 | Glycolysis |
| 428 | PSME1 | Q06323 | 2.3 | 1.76E-05 | 2.0 | 3.40E-05 | Immunoproteosome |
| 734 | TUBB2A | Q13885 | 0.42 | 1.79E-05 | 0.48 | 1.46E-06 | Cytoskeletal |
| 785 | UCHL1 | P09936 | 0.59 | 1.89E-05 | 0.55 | 9.13E-06 | Stabilises free ubiquitin |
| 544 | TAGLN3 | Q9U115 | 0.5 | 2.55E-05 | 0.5 | 4.52E-07 | Neuronal growth |
| 1046 | TUBB2C | P68371 | 0.45 | 2.59E-05 | 0.59 | 0.00113 | Cytoskeletal |
| 774 | PDXP | Q96GD0 | 0.33 | 2.62E-05 | 0.5 | 4.72E-05 | Phosphatase activity |
| 794 | PRDX3***** | P30048 | 1.9 | 2.72E-05 | *1.5* | *0.0276* | Antioxidant |
| 823 | HPRT1 | P00492 | 1.6 | 3.32E-05 | *1.0* | *0.596* | Purine synthesis |
| 379 | VDAC2 | P45880 | 0.71 | 3.67E-05 | *0.83* | *0.097* | Ion transport |
| 748 | NAPG | Q99747 | 0.45 | 4.70E-05 | 0.59 | 9.04E-06 | Vesicle transport |
| 459 | UCHL1 | P09936 | 0.77 | 4.77E-05 | *0.83* | *0.00707* | Stabilises free ubiquitin |
| 657 | UBE2N | P61088 | 2.2 | 5.20E-05 | *1.1* | *0.807* | Ubiquitination |
| 786 | SEPT11 | Q92599 | 0.59 | 6.54E-05 | 0.63 | 0.00129 | Vesicle transport |
| 828 | PRDX3 | P30048 | 0.66 | 7.37E-05 | 0.77 | 0.00058 | Antioxidant |
| 812 | PSME2 | Q9UL46 | 2.0 | 9.5E-05 | 2.0 | 0.00169 | Immunoproteosome |
| 822 | HSPD1 | P10809 | 0.63 | 9.5E-05 | 0.63 | 0.00173 | Chaperone |
| 1062 | HSPB1 | P04792 | 0.5 | 0.000106 | 0.71 | 0.00219 | Chaperone |
| 69 | DPYSL2 | Q16555 | 0.66 | 0.000118 | *0.77* | *0.0249* | Cytoskeletal |
| 285 | ACOT7 | O00154 | 0.5 | 0.000124 | 0.53 | 2.84E-05 | Acetyl-CoA binding |
| 605 | MBP* | P02686 | 0.53 | 0.000131 | *0.83* | *0.122* | Myelin |
| 116 | PHGDH | O43175 | 0.55 | 0.000132 | 0.53 | 0.00187 | Serine biosynthesis |
| 467 | GFAP | P14136 | 2.4 | 0.000148 | *1.4* | *0.0213* | Cytoskeletal |
| 483 | DCXR* | Q7Z4W1 | 2.7 | 0.000186 | *1.2* | *0.145* | Glucose metabolism |
| 868 | UCHL1 | P09936 | 0.66 | 0.000229 | *0.77* | *0.00514* | Stabilises free ubiquitin |
| 276 | IDH3A | P50213 | 0.48 | 0.000239 | 0.59 | 3.65E-06 | TCA cycle |
| 916 | CKB | P12277 | 0.66 | 0.000245 | 0.66 | 1.49E-05 | ATP homeostasis |
| 487 | TPI1 | D3DUS9 | 2.1 | 0.000251 | *1.1* | *0.426* | Glycolysis |
| 556 | PEBP1 | P30086 | 2.7 | 0.000255 | *2.0* | *0.00575* | Intracellular signalling |
| 718 | DCD | A5JHP3 | 0.37 | 0.000259 | 0.43 | 3.06E-06 | Phosphatase activity |
| 66 | DPYSL2 | Q16555 | 0.63 | 0.000316 | *0.77* | *0.0813* | Cytoskeletal |
| 92 | CCT6A | P40227 | 2.2 | 0.000391 | 2.0 | 0.000454 | Protein folding |
| 91 | HIST1H4A* | P62805 | 1.6 | 0.000479 | *1.4* | *0.0726* | Chromatin binding |
| 498 | GRB2 | P62993 | 0.63 | 0.000499 | *0.91* | *0.172* | Signal transduction |
| 273 | hCG_2002* | Q59GE1 | 0.71 | 0.000548 | 0.66 | 6.60E-05 | Neuron growth |
| 579 | DCD | A5JHP3 | 2.4 | 0.000596 | *1.4* | *0.0132* | Phosphatase activity |
| 62 | DPYSL2 | Q16555 | 1.7 | 0.000689 | *1.4* | *0.0203* | Cytoskeletal |
| 756 | ATP6V1E1 | P36543 | 0.71 | 0.000709 | 0.63 | 2.82E-05 | Energy metabolism |
| 437 | CLIC* | Q9Y696 | 2.4 | 0.000737 | 2.5 | 0.000155 | Ion transport |
| 288 | TUBB2A | Q13885 | 0.66 | 0.000745 | *0.77* | *0.0068* | Cytoskeletal |
| 731 | TF* | P02787 | 1.5 | 0.000749 | *1.3* | *0.159* | Iron transfer |
| 843 | PDIA3 | P30101 | 1.4 | 0.000751 | 1.3 | 0.00263 | Protein folding |
| 409 | HSPA5 | P11012 | 2 | 0.000810 | 1.8 | 0.00035 | Chaperone |
| 488 | APOA1* | P02647 | 0.55 | 0.000811 | 0.53 | 0.00026 | Lipid metabolism |
| 270 | ALDOA | P04075 | 0.71 | 0.000941 | 0.66 | 0.00124 | Glycolysis |
| 771 | GFAP* | P14136 | 1.8 | 0.000971 | 1.7 | 0.000953 | Cytoskeletal |
| 740 | HSPB1 | P04792 | 0.66 | 0.00101 | 0.71 | 0.00193 | Chaperone |
| 898 | GLUD1* | P00367 | 1.4 | 0.00107 | *1.2* | *0.326* | Glutamate turnover |
| 966 | ATP6V1B2 | P21281 | 0.59 | 0.00112 | *0.63* | *0.00927* | Energy metabolism |
| 263 | OvBr SEPT | Q9UHD8 | 0.66 | 0.00114 | *0.71* | *0.00407* | Cytoskeletal |
| 789 | hCG_2002 | Q59GE1 | 0.59 | 0.00115 | *0.71* | *0.00316* | Neuronal growth |
| 801 | SEPT11 | Q9NVA2 | 0.63 | 0.00124 | 0.66 | 0.00104 | Vesicle transport |
| 154 | SEPT11 | Q9NVA2 | 0.71 | 0.00133 | 0.63 | 0.00161 | Vesicle transport |
| 28 | GPD2 | P43304 | 1.9 | 0.00149 | *1.0* | *0.698* | Lipid metabolism |
| 207 | ACTR1B | P42025 | 0.63 | 0.00169 | *0.71* | *0.0083* | Cytoskeletal |
| 25 | HSPA8 | P11142 | 0.63 | 0.00187 | *0.67* | *0.0221* | Chaperone |
| 1012 | SRI | P30626 | 1.8 | 0.00191 | *1.2* | *0.219* | Calcium homeostasis |
| 516 | GSTP1 | P09211 | 1.3 | 0.00192 | 1.2 | 0.000867 | Free radical clearance |
| 342 | DKFZp686 | P07355 | 1.4 | 0.00193 | 1.9 | 0.00220 | Unknown |
| 572 | TAGLN3 | Q9U115 | 2.8 | 0.00200 | *1.5* | *0.004* | Neuronal growth |
| 81 | CAT | P04040 | 1.4 | 0.00218 | *1.4* | *0.0343* | Nucleotide binding |
| 434 | GSTO1 | P78417 | 1.5 | 0.00234 | *1.2* | *0.162* | Glutathione metabolism |
| 444 | ACOT7 | O00154 | 1.6 | 0.00239 | *1.3* | *0.0282* | Acetyl-CoA binding |
| 838 | PGAM1 | P18669 | 1.6 | 0.00249 | *1.0* | *0.441* | Glycolysis |
| 876 | ALAD* | P13716 | 1.6 | 0.00251 | *1.3* | *0.0295* | Haeme production |
| 324 | TUBB2B | Q9BVA1 | 0.77 | 0.00263 | *0.83* | *0.00781* | Cytoskeletal |
| 403 | GNB1 | P62873 | 0.63 | 0.00269 | *0.71* | *0.0614* | GPCR subunit |
| 829 | SNCG | A9XXE1 | *0.43* | *0.0333* | 0.38 | 7.58E-09 | Unknown |
| 945 | HIST1H4A | P62805 | *1.6* | *0.0191* | 1.6 | 1.79E-05 | Chromatin binding |
| 277 | ALDOA | P04075 | *0.91* | *0.154* | 0.71 | 3.98E-05 | Glycolysis |
| 401 | CLIC1 | O00299 | *1.5* | *0.0629* | 0.55 | 0.000135 | Ion transport |
| 1073 | UQCRFSL | P0C7P4 | *0.83* | *0.197* | 0.66 | 0.000261 | Unknown |
| 772 | PSMB7 | Q99436 | *1.5* | *0.0083* | 1.5 | 0.000261 | 20s proteosome |
| 564 | PEBP1 | P30086 | *1.0* | *0.774* | 0.71 | 0.000298 | Intracellular signaling |
| 840 | MAP2K1 | Q02750 | *0.83* | *0.0488* | 0.71 | 0.000313 | Intracellular signaling |
| 317 | LASP1 | Q14847 | *1.2* | *0.161* | 1.8 | 0.000322 | Cytoskeletal |
| 466 | - | - | *1.4* | *0.0088* | 1.7 | 0.000437 | - |
| 217 | GLUL | P15104 | *0.63* | *0.0086* | 0.55 | 0.000471 | Glutamine synthesis |
| 223 | SUCLA2 | Q9P2R7 | *0.77* | *0.130* | 0.55 | 0.000493 | TCA cycle |
| 299 | DDAH1 | O94760 | *0.71* | *0.00356* | 0.71 | 0.000548 | NO regulation |
| 227 | CKB | P12277 | *0.83* | *0.0221* | 0.77 | 0.000996 | ATP homeostasis |
| 601 | SOD1 | P00441 | *0.83* | *0.218* | 0.63 | 0.00102 | Antioxidant |
| 419 | PAFAH1B2 | P68402 | *1.2* | *0.258* | 1.5 | 0.00111 | - |
| 1028 | - | - | *1.1* | *0.360* | 1.5 | 0.00129 | - |
| 443 | - | - | *1.4* | *0.0345* | 1.9 | 0.00185 | - |
| 530 | PRDX1 | Q06830 | *1.2* | *0.156* | 1.5 | 0.00214 | Antioxidant |
| 653 | PRDX5 | P30044 | *0.91* | *0.132* | 0.77 | 0.00237 | Antioxidant |
| 845 | GLUL | P15104 | *0.71* | *0.00863* | 0.63 | 0.00238 | Glutamine synthesis |
| 375 | - | - | *0.67* | *0.00387* | 0.71 | 0.00257 | - |

**Supplementary Table 3: Proteins altered in young GBM and in old GBM and inclusion of data that fails to achieve statistical significance.** Proteins altered in young GBM (relative to young controls), and proteins altered in old GBM (relative to old controls). Significant protein changes are listed in black and match the data shown in Table 1 (p-values shown are prior to Bonferroni correction with a factor 3). The data ‘missing’ in Table 1 is shown in red and are values for protein changes that failed to reach the predetermined statistical threshold (ie. p<0.003). Spot ID provides a unique 2DGE spot identifier and is important because several proteins were identified in multiple spots, for example OXCT1 in spot 119 and spot 120. Proteins marked with an asterisk indicate a spot where a second protein (or occasionally more) is present at a level close to that of the listed protein. Blank protein IDs (for example spot 243) could not be identified. The protein accession numbers (Uniprot), magnitude of protein response and p-values (ranked according to changes in young GBM) are listed for each altered protein.
